# Total Mesorectal Excision with or without Lateral Pelvic Lymph Node Dissection in Rectal Cancer

**DOI:** 10.1155/2023/6653624

**Published:** 2023-12-26

**Authors:** Mohamed Yehia Elbarmelgi, Ahmed Mohamed Abdelaal, Osama Refaie, Mohamed Tamer, Ali Ahmed Shafik

**Affiliations:** ^1^General and Colorectal Surgery, Cairo University, Giza, Egypt; ^2^General Surgery, Cairo University, Giza, Egypt

## Abstract

**Results:**

Incidence of local recurrence was slightly higher in Group A (8.7%) than in Group B (4.3%) but was not statistically significant. There was no statistical significance between both groups regarding distant metastasis (8.7% in Group A and 13% in Group B). Urinary and sexual dysfunctions were higher in Group B (26.1%) compared to those in Group A (21.7%) but were not statistically significant. The incidence of lateral pelvic lymph node metastasis was 30.4%. Also, intraoperative blood loss was higher and operative time was longer in Group B which was statistically significant (*P* value <0.001).

**Conclusion:**

Our conclusion was that prophylactic addition of LPLD to TME was not associated with a statistically significant decrease in the risk of local recurrence or distant metastasis in patients with rectal cancer, although it was numerically better. LPLD is associated with longer operative time and higher intraoperative blood loss.

## 1. Introduction

Colorectal cancer is the third leading cause of death round the world with a rising incidence [1]. Patients presenting with lower rectal cancer where the lower margin is at or below the peritoneal reflection may develop metastases to lateral pelvic lymph nodes which are outside the surgical field of TME. A retrospective multicenter study in Japan reported that the incidence of lateral pelvic lymph node metastasis in patients with T3 or T4 lower rectal cancer was 18.1% [2].

Two pathways are involved in lymphatic drainage of the middle and lower rectum. The superior lymphatic drainage system runs along the inferior mesenteric artery and lateral lymphatic drainage runs along the internal iliac artery [3–5].

The different approaches to LPLD between the East and West originate from the concept that lateral pelvic nodes are considered regional according to Japanese literature while the Western world, according to the AJCC guidelines (AJCC 8^th^ edition), confirms pelvic lateral lymph node stations as remote stations. Japanese surgeons suggest performing TME with bilateral pelvic lymphadenectomy without neoadjuvant treatment, as they expect that the risk of intrapelvic recurrence decreases by 50% [4, 6].

## 2. Aim of Work

The primary objective of this study was to compare the incidence of local recurrence in patients who underwent TME with or without LPLN dissection in extraperitoneal rectal cancer. The secondary objective of this study was to compare the incidence of distant metastasis, intraoperative time, intraoperative blood loss, and urinary/sexual dysfunction between both groups.

## 3. Patients and Methods

### 3.1. Study Type

This was an observational study.

### 3.2. Study Settings

This study was carried out from June 2020 to November 2021, and follow-up was completed in February 2023 on patients with rectal cancer of both genders and from all age groups presenting with extraperitoneal mid- and low-rectal cancer (T3 and T4) presenting to the colorectal general surgery department, Cairo University hospitals.

### 3.3. Inclusion Criteria and Exclusion Criteria

**Figure 1:**
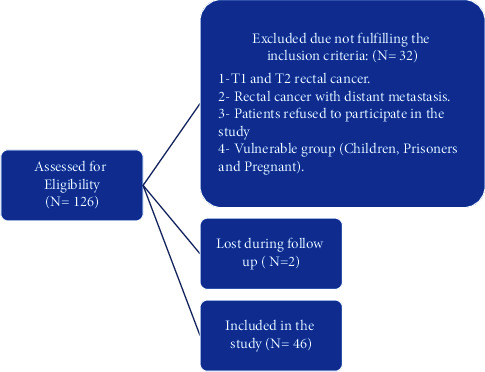
Flowchart showing exclusion and inclusion criteria.

Patients from both sexes and all age groups diagnosed with extraperitoneal rectal cancer (T3 and T4) who are candidates for neoadjuvant chemoradiation were included in the study.  Figure 1 shows the exclusion criteria plus patients who were recruited and then excluded from the study.

### 3.4. Preoperative Preparations

Ninety-two patients scheduled for surgery for rectal cancer were evaluated for the study. The patients were randomized using the closed envelope technique into two equal groups, each with 46 patients:

  Group (A) received preoperative neoadjuvant chemoradiation and TME was done
  Group (B) received no neoadjuvant chemoradiation and TME with LPLN dissection was done

Informed written consent was obtained from all individuals that participated in the study.

Group (A) was considered the control group from which the results of Group (B) were compared. Both groups were compared regarding demographic features and regarding comorbidities. Postoperative chemoradiation was administered in all cases according to the examined pathological specimen after resection.

Patients were assessed preoperatively by proper history taking and full clinical and digital rectal examination, colonoscopy, biopsy, MRI pelvis for locoregional staging, and CT for chest, abdomen, and pelvis to detect distant metastasis.

### 3.5. Preoperative Neoadjuvant Regimen

Neoadjuvant therapy involved 28 fractions totaling 50.4 Gy (45 Gy to the entire pelvis and 5.4 Gy to the tumor) over 5 weeks. This was supplemented with fluorouracil (5-FU) infusions at weeks one and five. Surgery was performed 6–8 weeks after the last chemoradiation session.

### 3.6. Surgical Procedure

**Figure 2:**
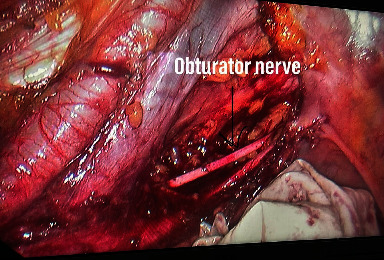
Lateral pelvic lymph node dissection.

Group A: patients who received preoperative neoadjuvant chemoradiation; TME was done. Group B: In addition to TME, the lateral pelvic lymph nodes in fatty and connective tissues were dissected after TME (Figure 2). All surgeries in both groups were done laparoscopically. Diversion loop ileostomy was done on all patients in whom bowel continuity was restored after completing postoperative adjuvant therapy.

### 3.7. Postoperative Follow-Up

Patients were referred to receive adjuvant chemoradiotherapy according to the stage and were followed up for 18 months to detect local recurrence. Follow-up is done monthly during a routine outpatient visit by clinical examination. MRI pelvis and CT chest and abdomen and pelvis are done every six months. Colonoscopy is done annually.

Both groups were compared regarding operative time, estimated intraoperative blood loss, incidence of local recurrence, incidence of distant metastasis (recurrence), histopathological examination of the surgical specimen, and urogenital complications (sexual and urinary dysfunctions). Urinary and sexual dysfunctions are assessed clinically during the follow-up visits, mainly erection, retrograde ejaculation, and urine retention or difficulty in micturition.

The manuscript was developed according to the STROBE checklist [7].

### 3.8. Statistical Analysis

Data were coded and entered using the Statistical Package for the Social Sciences (SPSS) version 28 (IBM Corp., Armonk, NY, USA). Data were summarized using mean, standard deviation, minimum, and maximum in quantitative data and frequency (count) and relative frequency (percentage) for categorical data. Comparisons between quantitative variables were done using the nonparametric Kruskal–Wallis and Mann–Whitney tests. For comparing categorical data, a chi-square (*χ*^2^) test was performed. The exact test was used instead when the expected frequency is less than 5. Correlations between quantitative variables were done using the Spearman correlation coefficient. *P* values less than 0.05 were considered statistically significant.

## 4. Results

Patients were compared on the following aspects.

### 4.1. Patients' Demographics

Table 1 shows the difference between both groups regarding demographic features including age and gender. There was no statistically significant difference between both groups regarding demographic features.

### 4.2. Comorbidities and ASA

Table 2 shows the distribution of diabetes mellitus, hypertension, and ASA grade between both groups; there was no statistically significant difference in all aspects.

### 4.3. Tumor Location

Table 3 shows the difference between both groups regarding tumor location. There was no statistical difference between either groups (*P* value = 0.225).

### 4.4. Pathological Type

Table 3 shows the difference between both groups regarding pathological types of tumors between both groups. There was no statistical difference between either groups (*P* value = 1).

### 4.5. Operative Time and Estimated Blood Loss

Table 4 shows the comparison between both groups regarding intraoperative blood time and estimated blood loss. Group B, who was subjected to a pelvic lymphadenectomy, showed more blood loss and operative time which was statistically significant (*P* value <0.001).

### 4.6. Local Recurrence and Distant Metastasis

The incidence of local recurrence was 8.7% in Group A and 4.3% in Group B (pelvic lymphadenectomy). Metastasis was in 4 patients in Group A (8.7%) and 6 patients in Group B (13.0%). However, there was no statistically significant difference between the 2 groups in both parameters. This is shown in Table 5.

All our patients have free distal, proximal, and circumferential margins which help compare local recurrence and distant metastasis rates.

### 4.7. Urinary and Sexual Dysfunctions and Postoperative Complications

We did not experience any mortality or major postoperative complications such as leakage (as all patients were diverted) or wound dehiscence. As for urinary and sexual dysfunctions, they occurred in 12 patients in Group B (26.1%) compared to 10 patients (21.7%) in Group A. This was statistically insignificant (*P* value = 0.730).

### 4.8. Mesorectal Lymph Nodes

There was no statistically significant difference between either groups in the number of retrieved and positive mesorectal lymph node. This is shown in Table 6.

### 4.9. Lateral Pelvic Lymph Nodes in Group B

The number of patients with a positive pelvic lymph node was 14 (30.4%). The mean number of retrieved pelvic lymph node in Group B was 12.04 ± 3.48 with a minimum of 2 and a maximum of 18 lymph nodes retrieved, while the mean of positive pelvic lymph node was (0.39 ± 0.14).

### 4.10. Relation between Recurrence and Pathological Type

Mucinous adenocarcinoma was the pathological type in 18 patients (*N* = 92, 19.5%), distant metastasis occurred in 6 of those patients (3.33%). Nonmucinous tumors were the pathological type in 74 patients (80.4%) and distant metastasis occurred in 4 patients (5.4%) which was statistically significant (*P* value = 0.044).

Regarding local recurrence, it occurred in 4 out of 18 patients with mucinous adenocarcinoma (22.2%) and in 2 out of 74 patients with nonmucinous adenocarcinoma (2.7%). The *P* value was 0.093; however, this was statistically insignificant.

## 5. Discussion

From the results of our study, LPLD showed no superiority over the routine neoadjuvant followed by TME strategy regarding distant metastasis, local recurrence, or number of retrieved lymph nodes but was also not associated by more urogenital consequences.

Regarding operative time, Hajibandeh et al. reported that TME with LPLD resulted in longer operative time (*P* < 0.00001) [8]. Also, Cribb et al. in 2021 reported that significantly longer operative times were observed in patients who underwent LPLD (*P*=0.008) [9]. In our study, operative time was greater in group B (LPLD) with significant difference.

Regarding estimated intraoperative blood loss, several studies reported more intraoperative blood loss in the lateral pelvic lymphadenectomy group and the difference was significant [9, 10]. In our study, operative time was greater in Group B (LPLD) which was also statistically significant.

As for local recurrence, Anania et al. in 2020 reported that the incidence of the overall local recurrence was not statistically different between the lateral pelvic lymph node dissection group and the nonlateral pelvic lymph node dissection group [4, 8]. Also, Ma et al. reported that there were no significant differences observed between both groups regarding local recurrence (*P*=0.077) [11, 12]. Tamura et al. reported a slight increase in local recurrence in TME without lateral pelvic lymphadenectomy compared to lateral pelvic lymphadenectomy, but it was not statistically significant. (21.4% vs. 14.8%, *P*=0.833) [13]. However, Fujita et al. in 2017 reported there was a statistical significance in the local recurrence rate in the TME with LPLN dissection (7.4%) compared to (12.6%) in TME alone groups (*P*=0.024) [2]. In our results, the incidence of local recurrence was 8.7% in Group A in and 4.3% in Group B (LPLD). Our results slightly favor lateral pelvic lymph node dissection, but there is no statistical significance.

Regarding distant metastasis, several studies reported that there was no statistically significant difference in metastasis between the dissection group and the nonlateral pelvic lymph node dissection group [4, 8, 11, 12]. However, Fahy et al. reported that the rate of metastasis was lower in the TME plus LPLN dissection group (27.3% versus 29.9%, respectively) (*P*=0.02) [14]. In our study, metastasis was seen in 4 patients in Group A (8.7%) while 6 patients in Group B (13%), there is no statistical significance in metastasis in both groups (*P* value = 1).

Regarding genitourinary complications, Wang et al. in 2020 reported that no statistically significant difference was observed regarding urinary complication (*P*=0.3) between patients who underwent LPLN dissection and those who did not, but with a higher possibility of sexual dysfunction [15]. Hajibandeh et al. reported that patients who underwent LPLN dissection had an increasing risk of urinary dysfunction (*P* < 0.00001) and sexual dysfunction (*P*=0.002) [8]. Anania et al. reported that the incidence of urinary dysfunction was significantly higher in the LPLN dissection patients group (37%) compared to the non-LPLN dissection patients' group (24.4%), while the incidence of sexual dysfunction was similar between both groups [4]. Also, Saito et al. in 2016 reported the incidences of sexual dysfunction in patients who underwent TME alone and TME with LPLN dissection were 68% and 79% (*P*=0.37) [16]. On the other side, Maeda et al. reported that only minor disturbances of bladder function were reported in 15% of patients who underwent LPLN dissection compared to only 25% of the nonpelvic lymph node dissection group, while regarding sexual dysfunction, the percentage was 27% in the LPLN dissection group compared to 20% in the other group who had partial or total impotency after surgery [17]. Also, Ito et al. demonstrated that urinary function would not be worse in cases where autonomic nerves were surgically preserved, even if LPLN dissection was performed for lower rectal cancer [18]. Our study revealed an increase in urinary and sexual dysfunction in Group B compared to Group A, but with no statistical significance between both groups.

There are a lot of variations in the incidence of lateral LN affection among the literature. Kobayashi et al. reported in their study in 2009 that the lateral pelvic LN involvement was 14.9%, while Ueno and his colleagues in 2005 reported that 17.9% had lateral pelvic lymph node metastasis [19, 20]. However, in 2021, Wang et al., in their study, reported much more patients with lateral pelvic LN disease reaching about 62.5% [21]. Lastly, Dev et al. in 2018 reported that the incidence of lateral pelvic LN metastasis in lower rectal cancer was 26.5% [22]. In our study, the incidence of lateral pelvic lymph node involvement was 30.4%.

Regarding the relation between recurrence and pathological type, Park et al. in 2015 reported that locoregional recurrence in colorectal cancers was 17.2% in the mucinous adenocarcinoma group and 13.7% in the nonmucinous adenocarcinoma group (*P*=0.451). Distant metastasis was 75.9% in the mucinous adenocarcinoma group and 81.1% in the nonmucinous adenocarcinoma group (*P*=0.329); these were statistically insignificant [23]. Numata et al. in 2012 reported that local recurrence in rectal cases and peritoneal dissemination were more frequently observed in patients with mucinous histology [24]. In our current study, there was no statistically significant difference between the mucinous and nonmucinous types regarding local recurrence, but there was a significant difference in distant metastasis in the mucinous adenocarcinoma type.

Lastly, whether you go for LPLN dissection or not, the most important issue for rectal cancer is to do total TME and to achieve clear distal, proximal, and circumferential surgical margins which can be achieved either by open, laparoscopic, robotic, or transanal surgery (TaTME) [25, 26].

## 6. Conclusion

Our conclusion was that prophylactic addition of LPLD in patients who do not have radiologically lateral LN affection to TME was not associated with a statistically significant decrease in the risk of local recurrence or distant metastasis in patients with rectal cancer. LPLD is associated with longer operative time and higher intraoperative blood loss.

### 6.1. Limitations of Study

A relatively small number of patients were included in the study. A larger number of patients are needed to assess and compare recurrence rates between both groups who underwent LPLD and those who did not in rectal cancer and to assess sexual and urinary dysfunctions. Also, a longer period of follow-up is needed to detect long-term outcomes and complications.

## Figures and Tables

**Table 1:** Demographic distribution.

		Mean	Standard deviation	*P* value
Age (years)	Both groups	47.7	13.08	0.216
	Group A (TME only)	44.09	11.94	
	Group B (LPLN)	51.35	13.41	

		Number	Percentage (%)	

Gender (female)	Group A (TME only) *n* = 46	20	43.5	0.359
	Group B (LPLN) *n* = 46	14	30.4	

**Table 2:** Comorbidity and ASA distribution.

		Group A		Group B		*P* value
		Count	%	Count	%	
DM	Yes	10	21.7	10	21.7	1
	No	36	78.3	36	78.3	

HTN	Yes	8	14.4	6	13.0	1
	No	38	82.6	40	87.0	

ASA	Group A	ASA 1…28 patients, ASA 2…10, ASA 3…8				1
	Group B	ASA 1…26 patients, ASA 2…8, ASA 3…12				

**Table 3:** Tumor location distribution and pathological type distribution.

		Group A		Group B		*P* value
		Count	%	Count	%	
Tumor location	Mid rectum	24	52.2	24	52.2	0.255
	Low rectum	18	39.1	10	21.7	
	Mid and low rectum	4	8.7	12	26.1	

Pathological type	Nonmucinous adenocarcinoma	38	82.6	36	78.3	1
	Mucinous adenocarcinoma	8	17.4	10	21.7	

**Table 4:** Operative time distribution and estimated blood loss distribution.

	Group A					Group B					*P*value
	Mean	SD	Median	Minimum	Maximum	Mean	SD	Median	Minimum	Maximum	
Operative time	173.91	22.56	172.00	135.00	212.00	233.57	31.30	230.00	170.00	310.00	<0.001
Blood loss	193.04	79.34	160.00	150.00	450.00	297.39	127.61	270.00	150.00	700.00	<0.001

**Table 5:** Local recurrence distribution and distant metastasis distribution.

		Group A		Group B		*P* value
		Count	%	Count	%	
Local recurrence	Yes	4	8.7	2	4.3	1
	No	42	91.3	44	95.7	

Metastasis	Yes	4	8.7	6	13.0	1
	No	42	91.3	4	87.0	

**Table 6:** Retrieved and positive mesorectal lymph nodes.

	Group A				Group B				*P* value
	Mean	SD	Minimum	Maximum	Mean	SD	Minimum	Maximum	
Mesorectal LNs	14.39	2.73	10.00	21.00	16.26	6.59	5.00	34.00	0.306
Positive mesorectal LNs	1.35	1.01	0.00	7.00	3.04	2.91	0.00	31.00	0.919

## Data Availability

The data used to support the findings of this study are available from the corresponding author upon reasonable request.

## Additional Points

*What Did This Study Add to Literature?* The main question of this study is to investigate the benefit of LPLD in the management of rectal cancer. LPLD is widely performed during the operation of low anterior resection of rectal cancer in East Asia (in Japan, for example); however, it does not have any popularity in the Western literature. The main objective of this study is to investigate its benefit on disease-free survival.

## Supplementary Materials

Supplementary Materials Supplementary file provided is the STROBE checklist.
